# A Small Intestinal Helminth Infection Alters Colonic Mucus and Shapes the Colonic Mucus Microbiome

**DOI:** 10.3390/ijms252212015

**Published:** 2024-11-08

**Authors:** Thomas C. Mules, Francesco Vacca, Alissa Cait, Bibek Yumnam, Alfonso Schmidt, Brittany Lavender, Kate Maclean, Sophia-Louise Noble, Olivier Gasser, Mali Camberis, Graham Le Gros, Stephen Inns

**Affiliations:** 1 Malaghan Institute of Medical Research, Kelburn, Wellington 6012, New Zealand; 2 Department of Medicine, University of Otago, 23A Mein St., Newtown, Wellington 6242, New Zealand; 3 Hugh Green Technology Centre, Malaghan Institute of Medical Research, Wellington 6012, New Zealand

**Keywords:** helminth, mucus, hookworm, intestinal barrier, trefoil factor, microbiome

## Abstract

Infecting humans with controlled doses of small intestinal helminths, such as human hookworm, is proposed as a therapy for the colonic inflammatory disease ulcerative colitis. Strengthening the colonic mucus barrier is a potential mechanism by which small intestinal helminths could treat ulcerative colitis. In this study, we compare C57BL/6 mice infected with the small intestinal helminth *Heligmosomoides polygyrus* and uninfected controls to investigate changes in colonic mucus. Histology, gene expression, and immunofluorescent analysis demonstrate that this helminth induces goblet cell hyperplasia, and an upregulation of mucin sialylation, and goblet-cell-derived functional proteins resistin-like molecule-beta (RELM-β) and trefoil factors (TFFs), in the colon. Using IL-13 knockout mice, we reveal that these changes are predominantly IL-13-dependent. The assessment of the colonic mucus microbiome demonstrates that *H. polygyrus* infection increases the abundance of *Ruminococcus gnavus*, a commensal bacterium capable of utilising sialic acid as an energy source. This study also investigates a human cohort experimentally challenged with human hookworm. It demonstrates that TFF blood levels increase in individuals chronically infected with small intestinal helminths, highlighting a conserved mucus response between humans and mice. Overall, small intestinal helminths modify colonic mucus, highlighting this as a plausible mechanism by which human hookworm therapy could treat ulcerative colitis.

## 1. Introduction

Intestinal helminths are multicellular organisms that have co-evolved with their hosts over millennia. Approximately two billion people are infected with intestinal helminths globally, the majority of whom are those living in countries with tropical and subtropical climates, which bring them into regular contact with infective larvae through contaminated soil. A high infection burden with intestinal helminths can have detrimental health effects, particularly in children, leading to significant investment in eradication programmes [1,2]. Conversely, data from epidemiological, preclinical, and, to a lesser extent, human clinical studies, have revealed that human hosts may also elicit benefits from infection with helminths, particularly in reducing the risk of autoimmune diseases such as the inflammatory bowel diseases ulcerative colitis and Crohn’s disease [2,3,4].

Infecting humans with controlled doses of small intestinal helminths such as human hookworm, termed hookworm therapy, is proposed as a therapy for ulcerative colitis [5]. Small intestinal helminths can modulate colonic inflammation through several mechanisms, including upregulation of the anti-inflammatory cytokine IL-10 and downregulation of the inflammatory cytokines IL-12, IFN-γ and TNF-α [6,7,8,9,10]. Another potential mechanism is helminths’ effect on colonic mucus, a continuous gel which protects the underlying epithelium from microbial antigens [2,11]. Patients with ulcerative colitis have an impaired mucus barrier which contributes to colonic inflammation, and strengthening the mucus layer is a potential therapy for ulcerative colitis [12,13,14].

Infection with helminths activates a type 2 immune response aimed at expelling the invading helminth characterized by the release of type 2 cytokines, IL-4, IL-5 and IL-13 [15,16]. This immune response induces mucus hypersecretion [17,18,19,20,21], increased production of goblet-cell derived functional proteins, such as mucins, resistin-like molecule-beta (RELM-β), and trefoil factors (TFFs) [19,20,22,23,24], and the enzymatic addition of sialic acid or sulphate residues to O-glycan chains on mucins [25,26,27,28]. These modifications result in mucus becoming more copious, viscous, and resistant to degradation, ultimately aiding with parasite expulsion, protection of the underlying epithelial layer, and maintenance of intestinal homeostasis [29]. So far, research characterizing the host’s mucus response to helminths has largely focused on mucus changes in the parasite’s intestinal niche. However, for changes in mucus induced by small intestinal helminths to protect against colonic inflammation, the colonic mucus also needs to be modulated.

From a clinical perspective, a potential therapeutic advantage of hookworm therapy over conventional therapies, which generally require daily dosing, is its ability to reside in the body for several years, meaning a single dose could potentially deliver a prolonged benefit without the need for repeat dosing [30]. To benefit from this characteristic, the immunological responses to human hookworm need to persist into the chronic phase of the infection. To date, the effect of helminths on mucosal responses in the chronic infection phase are poorly defined.

To address these gaps in knowledge, this study uses *Heligmosomoides polygyrus*, a helminth which exclusively resides in the small intestine and establishes a chronic infection in its host, to assess the effect of small intestinal helminths on colonic mucus in the acute and chronic phases of the infection [31]. Next, a cohort of humans experimentally challenged with the human hookworm *Necator americanus* is assessed to determine if there is conservation in the mucus response between humans and mice. This study reveals that a small intestinal helminth induces a site-specific, persistent, and predominantly IL-13 dependent mucus response in the colon, and that these changes may shape the colonic microbiome.

## 2. Results

### 2.1. H. polygyrus Induces Colonic Goblet Cell Hyperplasia

C57BL/6 mice were infected with *H. polygyrus*, and jejunal (site of infection), and colonic (intestinal site distant to the infection) tissue was collected to assess the intestinal mucus response. Goblet cell hyperplasia in the jejunum and colon was assessed by staining with Periodic-acid Schiff (PAS) and performing blinded image analysis to determine the percentage of PAS staining in standardised histology sections.

**Figure 1:**
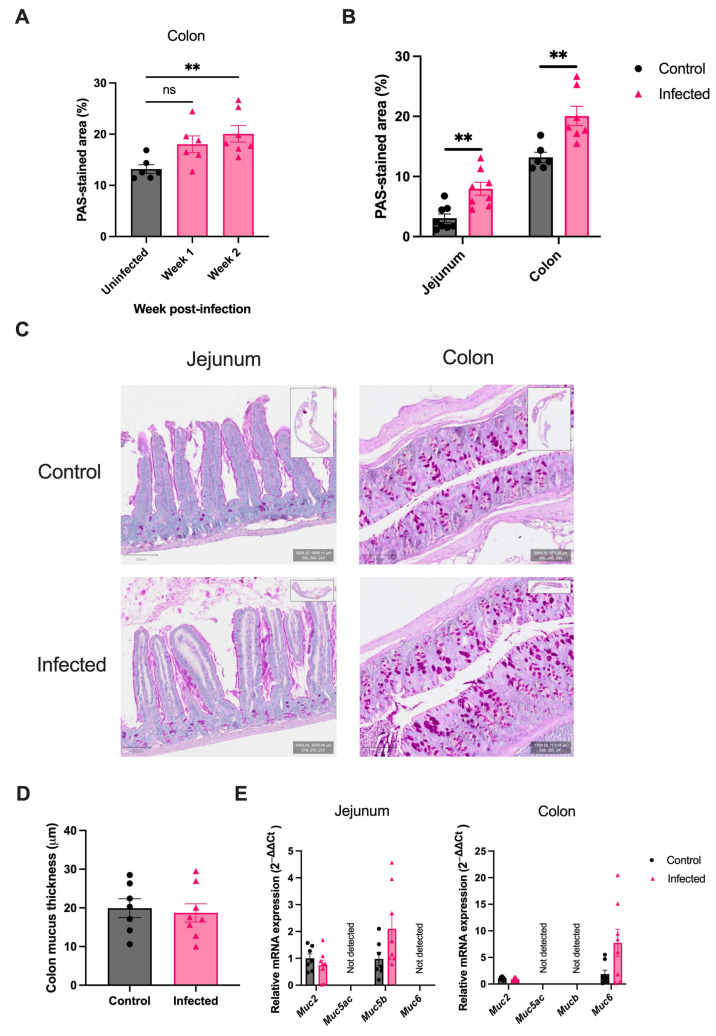
*H. polygyrus* induces pan-intestinal goblet cell hyperplasia. (**A**) Percentage of PAS-stained area in colonic sections from uninfected mice (black) and mice infected with 200 *H. polygyrus* larvae (pink) for one and two weeks, *n* = 6–7 from two experiments. (**B**) Percentage of sections positively stained by PAS in the jejunum and colon of uninfected mice and mice infected with *H. polygyrus* for two weeks, n = 6–8 from two experiments. (**C**) Representative images from Periodic acid Schiff (PAS) stained jejunum and colon from uninfected and infected mice. Goblet cells-stained magenta. Scale bar = 100 μm. (**D**) Colonic mucus thickness measured in PAS/Alcian Blue stained sections in uninfected and infected mice, *n* = 7–8 from two experiments. (**E**) Relative differences in mRNA expression of gel-forming mucins (Muc2, Muc5ac, Muc5b, and Muc6) in the jejunum and colon between uninfected and infected mice. *Rplp0* was used as a housekeeping gene. *n* = 8–10 from two experiments. Data were analysed with unpaired student *t*-test (**A**,**D**,**E**) or ANOVA with post-hoc Dunnett’s test (**C**). Mean ± SEM. ns, not significant; **, *p* < 0.01.

A significant increase in colonic goblet cells was observed two weeks post-infection, but was not observed at one week post-infection (Figure 1A). This meant the two-week time-point was used in further experiments to assess changes in intestinal mucus during this helminth’s acute infection phase. At two weeks post-infection, mice infected with *H. polygyrus* displayed goblet cell hyperplasia in the jejunum (PAS-stained area 6.8%, vs. 3.1%, *p* < 0.05) and colon (19.0%, vs. 13.2%, *p* < 0.05) when compared to uninfected age-matched controls (Figure 1B,C). These results highlight that an exclusively small intestinal helminth induces a pan-intestinal mucus response.

As goblet cells produce and secrete mucus, it was expected that the observed goblet cell hyperplasia would correlate with a thicker colonic mucus layer, however, no significant difference in mucus thickness was observed between infected and uninfected mice (18.7 µm vs. 19.9 µm, *p* = 0.794) (Figure 1D). Furthermore, there was no significant upregulation in mRNA expression of the gel-forming mucins (*Muc2*, *Muc5ac*, *Muc5b*, *Muc6*) in the jejunum or colon of *H. polygyrus*-infected mice (Figure 1E). In summary, no significant increase in mucus thickness or mucin expression was seen, despite the observed goblet cell hyperplasia.

### 2.2. H. polygyrus Upregulates Goblet-Cell Derived Proteins and Mucin Sialylation Throughout the Intestine

**Figure 2:**
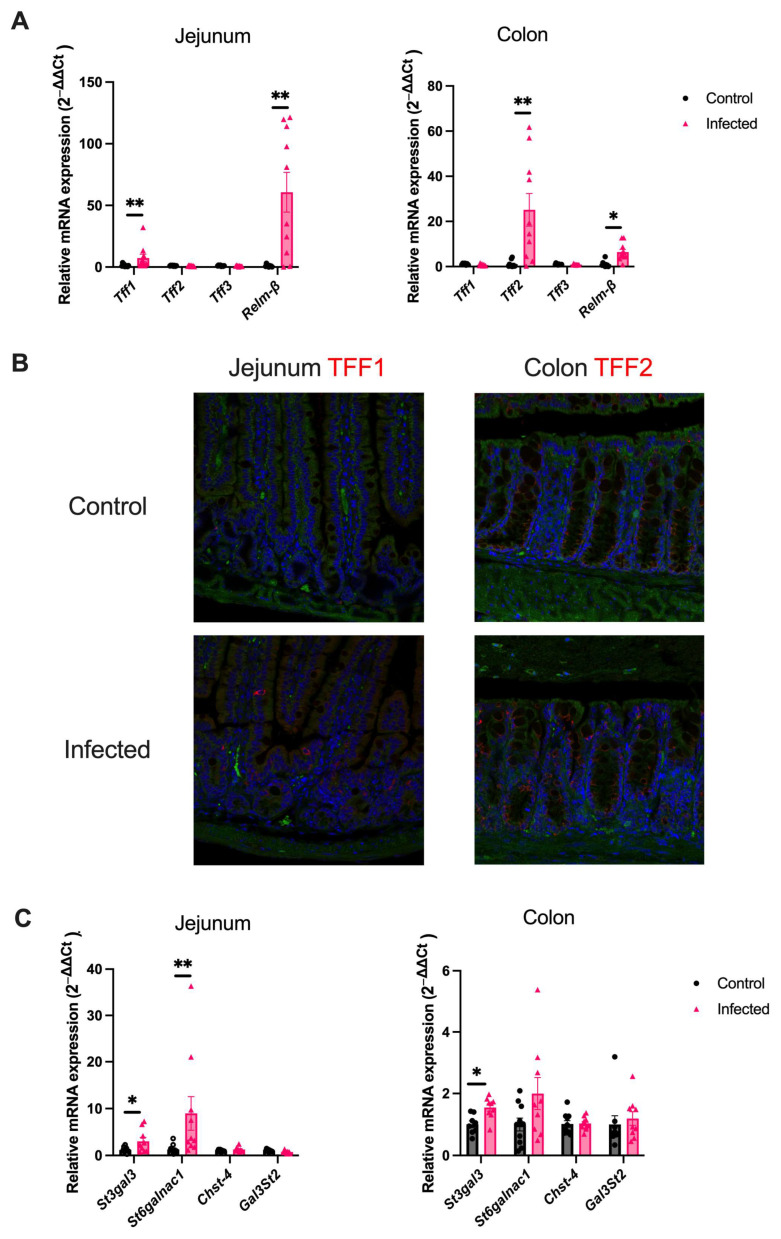
*H. polygyrus* induces pan-intestinal alterations in goblet cell-derived proteins and mucin sialylation. (**A**) Relative differences in mRNA expression of trefoil factors (*Tff1*, *Tff2*, and *Tff3*) and *Relm-β* in the jejunum and colon of uninfected mice and mice infected with 200 *H. polygyrus* larvae for two weeks, *n* = 8–10 from two experiments. (**B**) Representative immunofluorescence image staining for TFF1 (red) and TFF2 (red) in the jejunum and colon, respectively. DAPI = blue; green = autofluorescence. 40× magnification. (**C**) Relative differences in mRNA expression of sialyltransferases (*St3gal3* and *St6galnac1*) and sulphotransferases (*Chst-4* and *Gal3st2*) in the jejunum and colon of uninfected and infected mice, *n* = 8–10 from two experiments. *Rplp0* or *Gapdh* was used as a housekeeping gene. Data were analysed with unpaired student *t*-test. Mean ± SEM. *, *p* < 0.05; **, *p* < 0.01.

To investigate the mucus response further, attention shifted to other goblet-cell derived proteins that could be upregulated in response to a helminth infection. Trefoil factors (*Tff1*, *Tff2* and *Tff3*) and *Relm-β* mRNA expression were compared between mice acutely infected with *H. polygyrus* and age-matched controls. In the jejunum, there was a marked upregulation of *Tff1*, while no change in *Tff2* or *Tff3* was observed (Figure 2A). In contrast, the expression of *Tff2* significantly increased in the colon, while there was no change in *Tff1* or *Tff3* gene expression (Figure 2A). The divergent TFF response between the jejunum and colon was confirmed by staining for TFF1 and TFF2 using immunofluorescence (Figure 2B). To ensure a change in jejunal *Tff2* and *Tff3* mRNA expression did not occur at an earlier time-point in the infection, these genes were also assessed one week post-infection and no significant upregulation was observed. *Relm-β* mRNA expression was significantly upregulated in both the jejunum and colon in the acute phase of an *H. polygyrus* infection (Figure 2A).

*H. polygyrus* has been previously shown to increase the sialylation of mucin in the jejunum of infected mice [27]. This finding was confirmed in the current study by demonstrating a significant upregulation of the sialytransferases, *St3gal3* and *St*6*galnac1*, while no induction of sulphotransferases (*Chst-4* and *Gal*3*st*2) was observed. In addition, in the colon of infected mice, the sialyltransferase, *St3gal3*, was significantly upregulated, again with no change in the expression of sulphotransferases (Figure 2C).

### 2.3. Goblet Cell Changes Are Sustained into the Chronic Infection Phase

**Figure 3:**
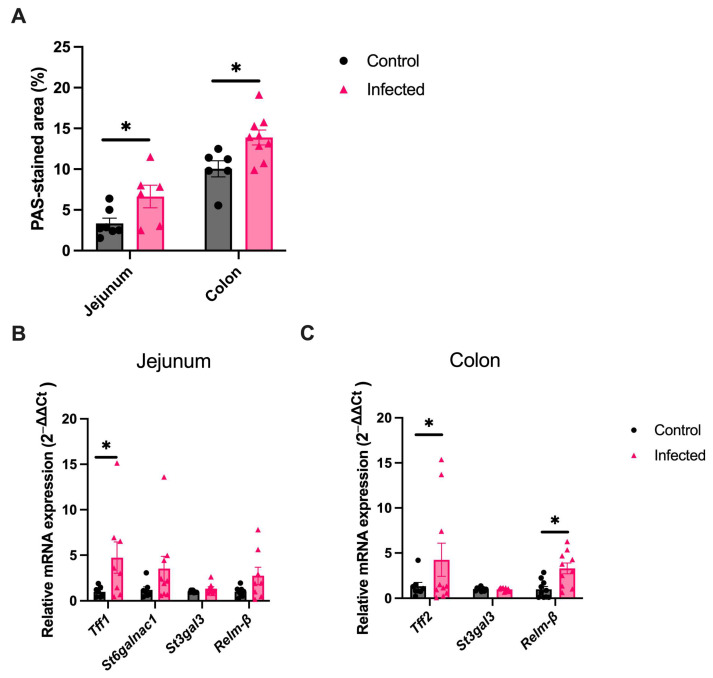
Goblet cell hyperplasia, upregulation of goblet-cell derived proteins, and colonic mucin sialylation are sustained in the chronic phase of an *H. polygyrus* infection. (**A**) Percentage of sections positively stained by PAS in the jejunum and colon of uninfected mice (black) and mice infected with *H. polygyrus* (pink) for six weeks, *n* = 6–9 from two experiments. (**B**,**C**) Relative difference in mRNA expression in the jejunum (**B**) and colon (**C**) between uninfected and infected mice six weeks post infection. Only genes demonstrating a significant difference in the acute infection phase were assessed. *n* = 8–10 from two experiments. *Rplp0* or *Gapdh* was used as a housekeeping gene. Data were analysed with unpaired student *t*-test. Mean ± SEM. *, *p* < 0.05.

In C57BL/6 mice, chronic infection is a typical feature of *H. polygyrus*, which enabled the assessment of whether the changes in mucus persisted during the chronic infection phase. First, an ongoing infection was confirmed in all infected mice at six weeks post-infection (mean intestinal worm burden = 13 worms, range 4–19). Goblet cell hyperplasia persisted in the jejunum (PAS-stained area 6.4%, vs. 3.4%, *p* < 0.05) and colon (13.9% vs. 10.0% *p* < 0.05) in the chronic infection phase compared to age-matched controls (Figure 3A). In the jejunum, *Tff1* expression remained significantly upregulated (Figure 3B), whilst in the colon *Tff2* and *Relm-*β remained significantly upregulated (Figure 3C).

### 2.4. H. polygyrus Modifies the Colonic Mucosa-Associated Microbiome

**Figure 4:**
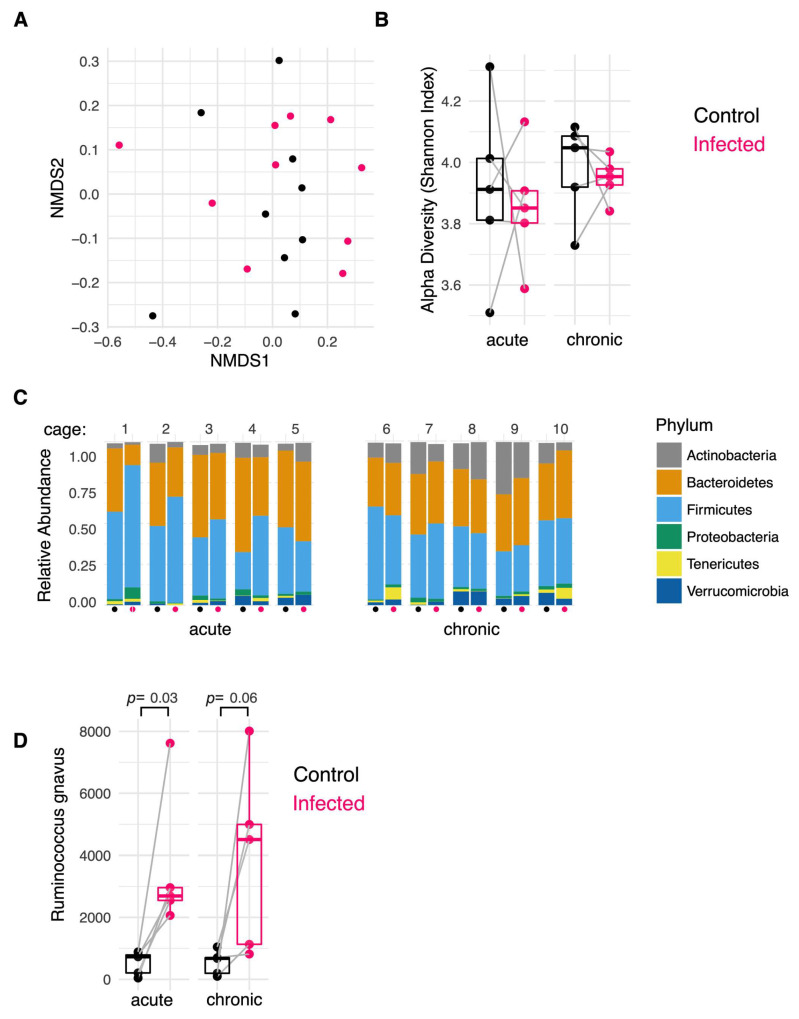
*H. polygyrus* induces a sustained alteration in the composition of the colonic mucosa-associated microbiome. (**A**,**B**) No significant differences in β-diversity (PERMANOVA, *p* > 0.05) (**A**) or α-diversity (paired Wilcoxon test, *p* > 0.05) (**B**) were observed between uninfected and infected cage-mates in the acute or chronic infection phase. Black dots = uninfected mice, pink dots = infected mice, grey line = co-housed mice (**C**) Relative abundances at the Phylum level were unchanged following infection (ANOVA, *p* > 0.05). (**D**) An increase in the abundance of the taxa *R. gnavus* was observed in the acute and chronic infection phases in mice infected with *H. polygyrus* (DESeq2). Data are from 5 mice/group from a single experiment.

The colonic mucus layer provides a natural habitat for particular gut microbes to reside in, often referred to as the mucosa-associated microbiota [32]. It was hypothesised that the observed changes in mucus composition induced in a chronic *H. polygyrus* infection would alter the composition of the colonic mucosa-associated microbiome. To assess for this, the colonic mucosa-associated microbiome in infected and uninfected mice was compared at the acute and chronic infection timepoints. Overall, no significant differences in β-diversity or α-diversity were observed between the uninfected and infected cohoused mice at these two timepoints (Figure 4A,B). In addition, no differences in the relative abundances at the Phylum level were observed (Figure 4C). When the abundances of individual operational taxonomic units (OTUs) were compared, an increase in the abundance of an OTU classified as *Ruminococcus gnavus* was seen in both the acute (*p* = 0.03) and chronic (*p* = 0.06) infection phases in all infected mice relative to their uninfected cage-mates (Figure 4D). Of relevance, *R. gnavus* possess sialidases which allow it to cleave sialic acid from mucin O-glycan chains to be used as an energy source. Therefore, the proliferation of this taxon is likely due to the increased colonic mucin sialylation induced in an *H. polygyrus* infection.

### 2.5. H. polygyrus Transiently Alters Mucus Composition in the Lung

**Figure 5:**
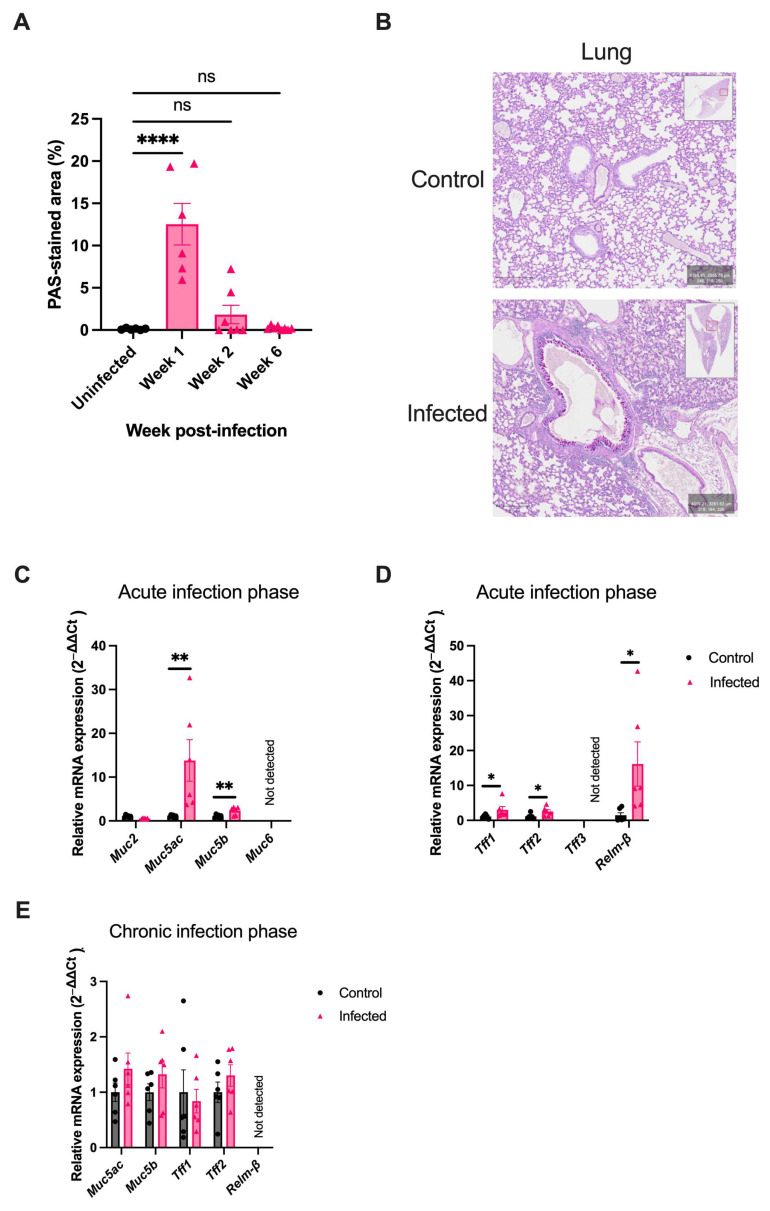
*H. polygyrus* induces goblet cell hyperplasia and upregulates goblet-cell-derived proteins in the lung. (**A**) Percentage of PAS-stained area in lung sections from uninfected mice (black) and mice infected with *H. polygyrus* (pink) for one, two, and six weeks, *n* = 6 per timepoint from two experiments. (**B**) Representative images from PAS-stained lung from mice one week post-infection, and uninfected mice. Goblet-cells-stained magenta. Scale bar = 100 μm. (**C**,**D**) Relative differences in mRNA expression in the lung between uninfected mice and mice infected with *H. polygyrus* for one week (acute infection phase), *n* = 6 from two experiments. (**E**) Relative differences in mRNA expression in the lungs of uninfected and mice infected with *H. polygyrus* for six weeks (chronic infection phase). Only genes demonstrating a significant difference in the acute infection phase were assessed. *n* = 6 from two experiments. *Rplpo0* or *Gapdh* was used as a housekeeping gene. Data were analysed with unpaired student *t*-test (**C**–**E**) or ANOVA with post-hoc Dunnett’s test (**A**). Mean ± SEM. ns, not significant; *, *p* < 0.05. **, *p* < 0.01, ****, *p* < 0.0001.

To determine if the mucus response induced by *H. polygyrus* only occurs in the intestine or also occurs in distant organs, the mucus response in the lung was examined. *H. polygyrus* is a strictly enteric helminth, so does not transit the lung in its lifecycle [31]. Despite not transiting the lung, goblet cell hyperplasia was observed in the lung of infected mice. In contrast to the pattern of goblet cell hyperplasia observed in the intestine, a significant increase in the PAS-stained area was only observed at one week post-infection (12.5% vs. 0.1%, *p* < 0.001), and was not sustained in the chronic infection phase (0.2% vs. 0.1%, *p* = 0.141) (Figure 5A,B). In the lung, a significant upregulation in the mRNA expression of the gel-forming mucins, *Muc5ac* and *Muc5b* (Figure 5C), trefoil factors, *Tff1* and *Tff2*, and *Relm-*β (Figure 5D), was observed in the acute infection phase. In keeping with the resolution of goblet cell hyperplasia in the chronic infection phase in the lung, the upregulation of goblet-cell-derived proteins was not sustained in this organ (Figure 5E).

### 2.6. IL-13 Orchestrates the Helminth-Mediated Systemic Mucus Response

**Figure 6:**
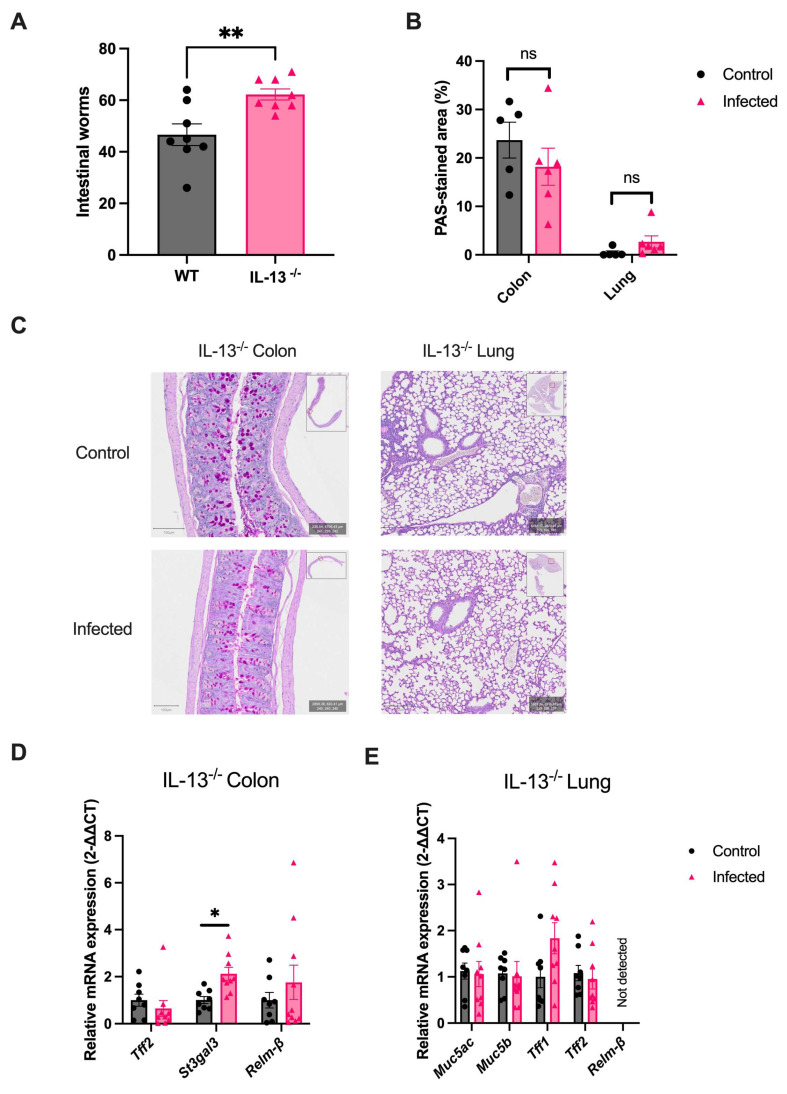
IL-13 drives mucus responses at sites distant to the intestinal niche of *H. polygyrus*. (**A**) Intestinal helminth burdens in wild-type (WT) and IL-13 knockout (IL-13^−/−^) mice two weeks after being infected with 200 *H. polygyrus* larvae, n = 8 mice/group from a single experiment. (**B**) Percentage of sections positively stained by PAS in the colon and lung of uninfected and infected IL-13^−/−^ mice in the acute infection phase (two weeks post-infection for colon and one week post-infection for lung), n = 5–7 from two experiments. (**C**) Representative images from PAS-stained colon and lung from uninfected and infected IL-13^−/−^ mice. Goblet cells are stained magenta. Scale bar = 100 μm. (**D**,**E**) Relative differences in mRNA expression in the colon (**D**) and lung (**E**) between uninfected and infected IL-13^−/−^ mice. Only genes demonstrating a significant difference in the acute infection phase in WT mice were assessed. RPLP0 or GAPDH was used as a housekeeping gene. N = 8–10 from two experiments. Data were analysed with unpaired student *t*-test. Mean ± SEM. ns, not significant; *, *p* < 0.05; **, *p* < 0.01.

It was previously demonstrated that the Th2 effector cytokine, IL-13, is the predominant driver of the local intestinal mucus response in a helminth infection [33,34,35]. To assess whether IL-13 also directs this response at sites distant to the infection, IL-13 knockout mice on a C57BL/6 background (*IL-13*^−/−^) were infected with *H. polygyrus* and the mucus response in the colon and lung was assessed. A significant difference in intestinal worm burdens between *IL-13^−/−^* and wild-type (WT) mice at two weeks post-infection (62 vs. 47 worms, *p* < 0.01) was observed (Figure 6A). The absence of IL-13 caused ablation of most mucus changes observed in WT mice acutely infected with *H. polygyrus*, including goblet cell hyperplasia in the colon and lung (Figure 6B,C); upregulation of *Tff2* and *Relm-*β in the colon (Figure 6D); and upregulation of mucins (*Muc5b* and *Muc5ac*) and Tff2 in the lung (Figure 6E). *St3gal3* expression remained upregulated in the colon of *IL-13^−/−^* mice.

### 2.7. Trefoil Factor 1 Is Upregulated in Humans Experimentally Challenged with Human Hookworm

TFFs can be measured in human serum and stool meaning changes during a helminth infection could be assessed in the absence of intestinal biopsies [36,37]. To assess if a similar trefoil factor response occurs in humans chronically infected with helminths, healthy volunteers were experimentally challenged with the human hookworm *N. americanus*, and changes in serum and faecal TFF1 and TFF2 were assessed. The human cohort enrolled in this study has been described previously [30]. Briefly, the cohort consisted of thirteen hookworm-naive healthy volunteers, with a median age of 47 years (range 19–64), and 54% were female. Participants were infected with 30 infective L3 larvae and followed up for 48 weeks post-infection. Blood and stool samples were collected at regular intervals (every 4–12 weeks), and a viable infection was confirmed by quantifying faecal egg counts throughout the follow-up period. Intestinal worm burden was assessed at 20 weeks post-inoculation using capsule endoscopy. One participant withdrew from the study at week 6 post-infection and was excluded from the analyses.

**Figure 7:**
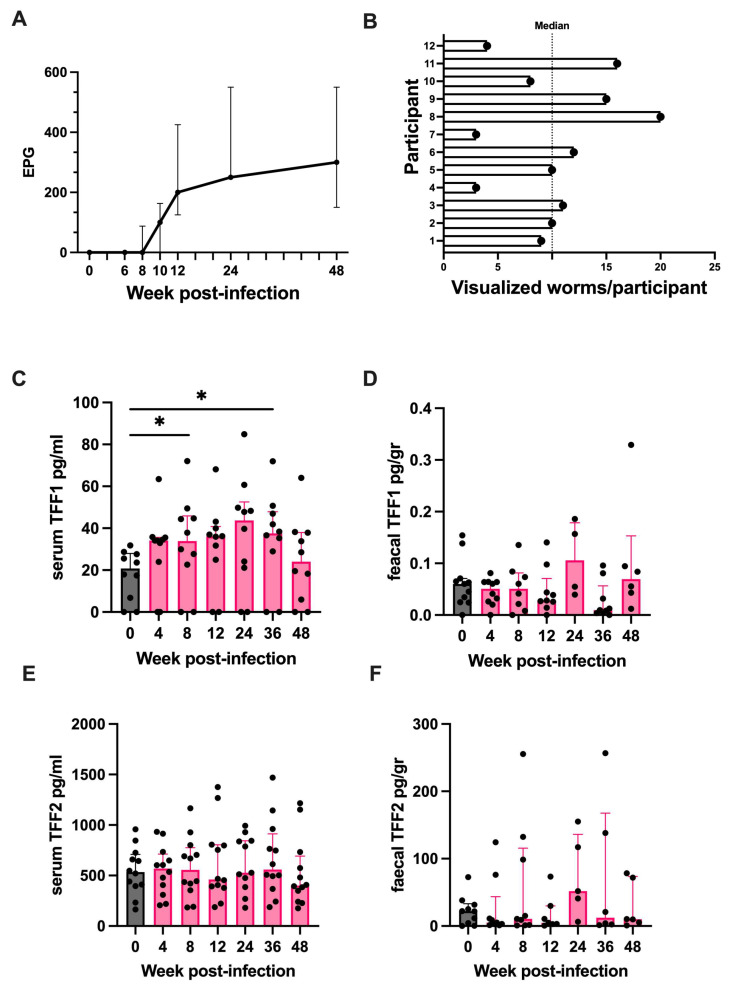
Serum TFF1 increases in humans experimentally challenged with human hookworm. (**A**) Median faecal egg counts in 12 healthy participants experimentally challenged with the human hookworm, *N. americanus*, during the 48-week follow-up period. (**B**) Number of visualised intestinal hookworm using capsule endoscopy at 20 weeks post-infection, line = median. (**C**–**F**) ELISA results showing concentrations of human serum TFF1 (**A**), faecal TFF1 (**B**), serum TFF2 (**C**), and faecal TFF2 (**D**) at baseline and 4, 8, 12, 24, 36, and 48 weeks post-infection. n = 12. Single outlying participant removed from (**C**) to improve data visualisation (data including outlier displayed in Figure S1A. Data were analysed using Friedman test with Dunn’s post-hoc multiple comparisons test. Median and IQR. Significant changes compared to baseline levels are indicated with an asterisk. *, *p* < 0.05.

All participants had a viable infection throughout the 48-week follow-up period (median faecal egg count 48 weeks post-infection = 300 eggs/g, range 100–550; and median visualised worms per participant at 20 weeks post-inoculation = 10 worms, range 3 to 20) (Figure 7A,B). A statistically significant increase in serum TFF1 was observed (*p* < 0.01). Compared to baseline levels, the median concentration of serum TFF1 was significantly elevated at week 8 (22.1 pg/mL vs. 33.9 pg/mL, *p* < 0.05) and week 36 (22.1 pg/mL vs. 37.5 pg/mL, *p* < 0.05) post-infection (Figure 7C). No significant change in faecal TFF1 (Figure 7D), serum TFF2 (Figure 7E), or faecal TFF2 (Figure 7F) was observed compared to baseline levels (*p* > 0.05 for all comparisons).

## 3. Discussion

The host’s immune response to a small intestinal helminth infection involves modifying the constituents of mucus to enhance its physiochemical properties, aid with helminth expulsion, and protect the underlying epithelial layer. While changes in the characteristics of mucus at a helminth’s intestinal niche are well known, the impact on mucus at distant intestinal sites is less clear. This study assessed the effect of small intestinal helminths on colonic mucus to determine if modification of colonic mucus is a plausible mechanism by which hookworm therapy could be harnessed to treat ulcerative colitis. It demonstrates that the exclusively small intestinal helminth *H. polygyrus* induces a predominantly IL-13-mediated effect on colonic mucus which involves the upregulation of functional mucus-associated proteins and mucin sialylation. A similar mucus response was also found in the lungs of infected mice, indicating an indirect impact on distal intestinal and extra-intestinal mucus. Next, this study found an increase in the abundance of *R. gnavus*, a commensal mucosa-associated bacterium which cleaves and utilises sialic acid as an energy source, in the colonic mucus of *H. polygyrus* infected mice. This observation demonstrates that helminths, such as *H. polygyrus*, may modulate the intestinal microbiome by upregulating mucin sialylation throughout the intestinal tract. Finally, consistent with this study’s preclinical data, humans experimentally challenged with human hookworm experienced a chronic increase in serum TFF1.

The mucus layer forms an important component of intestinal barrier function by limiting contact between luminal microbes and the epithelial layer [38]. The demonstrated changes in mucus composition local and distant to the site of infection are likely to improve the function of mucus by reducing the ability of microbes and microbial antigens to penetrate the mucus layer. For example, increased mucin sialylation makes mucus more viscous, TFFs increase mucus stability, and RELM-β has anti-microbial properties [39,40,41,42]. Hookworm therapy is proposed as a treatment for several diseases linked to defects in intestinal barrier function and the mucus layer, such as ulcerative colitis [5]. It is plausible that hookworm therapy could treat these diseases by altering mucus composition and improving intestinal barrier function [2,43].

The current study has demonstrated a sustained upregulation of TFF1 in the jejunum of *H. polygyrus* infected mice, and in the serum of humans experimentally challenged with human hookworm. The role of TFF1 in the host’s immune response against helminths has not been previously examined. The predominant site of TFF1 production in the body is the stomach. In the stomach, TFF1 promotes mucosal healing by enhancing cell migration, inhibiting apoptosis, and promoting angiogenesis, and plays an important role in healing mucosal damage, such as gastric ulceration, and in suppressing gastric tumour formation [44]. Although there has been limited research into its role in the intestinal tract, TFF1 is observed to increase at sites of intestinal inflammation, such as in patients with active IBD, and in some non-intestinal inflammatory conditions, such as pancreatitis [45,46,47,48]. It is possible that the upregulation of TFF1 in a helminth infection is to assist with the healing of damaged mucosa caused by the invading helminth and the hosts’ immune response against the helminth.

Although the current study found a significant increase in serum TFF1 in infected humans, the source of TFF1 is unclear. A small amount of TFF1 is known to undergo endocrine secretion, typically from lymphoid organs and some solid organs (including the brain, thyroid, and pancreas), meaning it is possible that the source of elevated TFF1 is extra-intestinal [49]. This study attempted to address this question by measuring levels of TFF1 in the faeces from the same individuals; no significant change in faecal TFF1 was observed, indicating the source may be extra-intestinal. Taken together, these preclinical and clinical data suggest the TFF response in humans and mice chronically infected with intestinal helminths is conserved, and the role of TFF1 in helminth immunity warrants further exploration.

The intestinal mucus layer provides a natural habitat for particular gut microbes to reside in, often referred to as the mucosa-associated microbiota [32]. Mucin glycans serve as attachment sites for certain bacteria which possess carbohydrate-binding molecules (CBMs) promoting their colonisation. In addition, a group of microbes, known as mucin-degrading bacteria, express enzymes able to cleave glycans, allowing the bacteria to use the released glycans as a consistent source of energy. These glycan-degrading enzymes, known as glycosyl hydrolases (GHs), include sialidases, fucosidases, and sulphatases. It is postulated that the ability to cleave and metabolise mucin glycans in part determines the composition of the mucosa-associated microbiome [50,51]. This study has demonstrated a significant increase in abundance of the taxa *R. gnavus* in the colonic mucosa-associated microbiome following infection with *H. polygyrus*. *R. gnavus* is a Gram-positive anaerobic bacterium belonging to the phylum Firmicutes. This bacterium harbours CBM40, allowing it to bind to mucin glycan chains and GH33 so it can cleave terminal sialic acid residues to utilise sialic acid as a source of energy [50,51]. It is reasonable to conclude that the increase in colonic mucin sialylation induced by *H. polygyrus* provides an optimal environment for *R. gnavus* to proliferate.

There is significant interest in the role of *R. gnavus* in disease pathogenesis or prevention. Although present in a healthy human microbiome, *R. gnavus* is disproportionately represented in the microbiome of patients affected by a range of diseases including inflammatory bowel disease, irritable bowel syndrome, and metabolic syndrome [52,53,54]. It is still to be determined if *R. gnavus* plays a role in the pathogenesis of these diseases or simply benefits from the disease-related changes in the intestine which could favour its expansion [55]. Conversely, the proliferation of *R. gnavus* could benefit its host by increasing SCFA production and promoting tryptophan metabolism [55,56]. Finally, the proliferation of *R. gnavus* could be beneficial to the host by occupying the glycan chain binding sites, which impedes the adhesion of certain pathogenic bacteria which also possess CBMs and GHs [57]. Further research to determine the role of *R. gnavus* in disease may provide further insight into helminths’ impact on the health of the host.

The identified changes in colonic mucus suggest small intestinal helminths strengthen the colonic mucus barrier, however, mucus penetrability was not assessed. The examination of mucus penetrability should be made a priority of future research [12]. Future studies should also include the quantitative analysis of protein expression, rather than only assess changes in mRNA expression. Another limitation of this study was only examining the human immune response in the peripheral circulation and fecal samples, which is not always reflective of immunological changes in infected tissues. Assessing mucosal responses during a controlled hookworm infection will require serial intestinal biopsies.

## 4. Materials and Methods

### 4.1. Mice and H. polygyrus Infection

All animal procedures were approved by the Victoria University Animal Ethics Committee, Wellington, New Zealand. Specific-pathogen-free C57BL/6 and IL-13 knockout (C57BL/6 background) mice were bred and housed at the Biomedical Research Unit, Malaghan Institute of Medical Research, Wellington, New Zealand. *IL-13^−/−^* mice were generated by the Australian Phenomics Network and Monash Genome Modification Platform, Melbourne, Australia. All the mice used in this study were 8- to 10-week-old female mice. Mice were maintained on standard rodent chow and acidified water ad libitum, with a 12-h light/dark cycle.

*Heligmosomoides polygyrus* infective L3 larvae were generated in the laboratory based on previously described methods [58]. Briefly, faeces from infected mice were mixed with charcoal and water, and incubated at 26 °C for 1 week. The larvae were recovered using a modified Baermann apparatus, washed with saline, and stored at 4 °C until infection. The motility of L3 larvae was assessed prior to inoculation to check viability. Mice were orally infected with approximately 200 larvae by gavage. Worm burdens were assessed by counting the number of worms present in the proximal small intestine using the Baermann technique [58]. Briefly, the proximal half of the small intestine was removed, opened longitudinally, and incubated in phosphate-buffered saline for 12 h before collecting and counting adult worms

Mice were euthanised and dissected at 1 week or 2 weeks post-infection to analyse changes in the acute infection phase in the lung and intestine, respectively, while 6 weeks post-infection was used to analyse changes in the chronic infection phase. Age-matched uninfected mice were used as controls.

### 4.2. Human Experimental N. americanus Infection

The human cohort used in the current study has been previously described and was approved by the New Zealand Health and Disability Ethics Committee (Ethics reference 19/CEN/81) [30]. In summary, thirteen healthy individuals were infected with 30 *N. americanus* larvae, followed-up for 48 weeks post-infection, and serum and faecal samples were taken every 4 to 12 weeks. One participant withdrew at 6 weeks post-infection and was excluded from the analyses.

A viable hookworm infection was assessed using two techniques: the detection of *N. americanus* eggs in the faeces of infected individuals using the McMaster technique, and hookworm visualization in the small bowel using the PillCamTM SB 3 Capsule Endoscopy System (Medtronic), as previously described [30].

### 4.3. Histology and Immunofluorescence Microscopy

A 1-cm segment of mid-jejunum, mid-colon, or entire right lung were fixed in 10% neutral buffered formalin or Carnoy’s solution (colon only) and embedded in paraffin using standard histological techniques. Five μm paraffin sections were cut using a paraffin microtome, attached to glass slides, and incubated overnight at 70 °C.

Formalin-fixed sections were deparaffinised in stepwise baths of xylene, hydrated in decreasing concentrations of ethanol, and then stained with Periodic-acid Schiff (PAS). Colonic sections fixed in Carnoy’s solution were deparaffinised and hydrated in stepwise baths of xylene, methanol, and ethanol, and then stained with PAS/Alcian blue. For immunofluorescent staining, jejunal and colonic formalin-fixed sections were stained for TFF1 (PA5-95930, ThermoFisher Scientific, MSA, Waltham, MA, USA) or TFF2 (BS-1921R, ThermoFisher Scientific, MSA, USA), followed by goat-anti-rabbit Alexa 555 conjugated antibody (A32732, ThermoFisher Scientific, MSA, USA), using standard indirect immunofluorescence techniques.

### 4.4. Quantification of Histological Staining

Whole slide images were captured using a whole slide scanner VS200 (Olympus, Tokyo, Japan) equipped with UPlanSApo 20X N.A. 0.75 objective and Hamamatsu ORCA-Flash 4 V3 sCMOS camera. Images were analysed using QuPath software version 0.3.0. by a blinded researcher [59]. Immunofluorescence images were obtained using an Olympus inverted microscope IX83 equipped with Laser Scanning Confocal Microscope head (FV3000, Tokyo, Japan) equip with a 405 nm (50 mW), 488 nm (20 mW), 561 nm (20 mW) excitation laser lines and highly sensitive detectors configurated with emission bands of 430–470, 500–540 and 570–620 respectively. All the images were captured using an UPLSAPO 40X N.A. 1.4 objective, with a 170 nm pixel size in image of 10 megapixels.

PAS staining was quantified in jejunal, colonic, and lung sections. In jejunal sections, the entire epithelial layer was selected; in colonic sections, at least 4 comparable longitudinally orientated areas, including at least 10 villi or crypt/gland structures, were selected per section; and in lung sections, ten transversely sectioned bronchioles were randomly selected, and the epithelial layer was annotated. Next, ten positive and negative PAS-stained regions were identified, and a pixel classifier algorithm was applied using a Random Trees Classifier with 50 as the maximum number of trees in a 2.19 µm/px resolution. Once results were consistent across several tissue sections, the algorithm was saved and applied to the entire data set in a batch image analysis process. The percentage of the PAS-stained area was determined for each section (1 section/mouse) (Figure S1B–D).

To quantify mucus thickness in colonic sections stained with PAS/Alcian blue, the mean diameter of the mucus layer was determined by measuring the mucus diameter perpendicular to the epithelial layer in at least 20 locations per section, as previously described (Supplementary Figure S1E) [60].

### 4.5. RNA Isolation and Real-Time qPCR

RNA was isolated from approximately 0.5 cm of mid-jejunum or mid-colon, or 30 mg of homogenised left lung, using RNeasy Mini Kit (Qiagen, Hilden, Germany). The quality and concentration of the RNA was assessed via Nanodrop (NanoDrop One C, ThermoFisher Scientific, MSA, USA). Then, 2000 ng of total RNA was used for cDNA synthesis using High-Capacity RNA-to-cDNA kit (Applied Biosystems, Waltham, MA, USA, ThermoFisher Scientific, MA, USA). Quantitative real-time PCR was performed using SYBRTM Green PCR Master Mix or TaqMan^®^ Gene Expression Assay platform (Applied Biosystems, ThermoFisher Scientific, MA, USA) using the Real-Time PCR System QuantStudio 7 instrument (Applied Biosystems) according to the manufacturer’s instructions. Forward and reverse primer sequences are displayed in Table S1. Gene expression was performed in duplicate, normalised against the housekeeping gene RPLP0 or GAPDH, and expressed as fold difference to uninfected age-matched mice following the 2^−ΔΔCt^ method.

### 4.6. Microbiota Analysis

Following weaning, mice were co-housed in cages of 5 mice. Mice were rotated between cages every 3 days until 8 weeks old to help normalise the microbiome between mice. Pairs of mice were then co-housed, and one mouse was infected with 200 *H. polygyrus* larvae, whilst the other mouse was the uninfected control. Ten mice (5 infected and 5 control) were euthanised at 2 weeks post-inoculation (acute infection phase) and 6 weeks post-inoculation (chronic infection phase). Mucus was collected from colon tissue by gently scraping the mucus from the surface of the colon. Genomic DNA was extracted using Qiagen DNeasy Power Soil Kit, Qiagen Inc., Germantown, MD, USA. The V3-V4 region of the 16S rRNA gene was amplified using barcoded primers for use on the Illumina platform. Sequence data are available at Dryad: https://datadryad.org/stash/share/NwLmRsMW0cQAwnV3hjCU-N2DKwZ-CYSCRS_9Y3ETIuI (accessed on 1 November 2024). Sequencing was performed using 300-base paired-end chemistry on an Illumina MiSeq instrument. Sequence data were trimmed, quality filtered, and clustered at 97% identity into Operational Taxonomic Units (OTUs) [61] following the mothur SOP pipeline [60]. To determine the taxonomic identity of OTUs, representative sequences were assigned taxonomic classification using a Bayesian classifier against the 16s rRNA gene database, Green Genes [62]. Before filtering, alpha diversity (Shannon index) was calculated and plotted using Phyloseq version 1.47 [63]. Data were transformed into relative abundance [x/sum(x)]. Differentially abundant taxa were identified using DESeq2 version 1.50 [64]. Data were further analysed in R version 4.3.1 using custom scripts and plotted using ggplot2 version 3.5.1 [65].

### 4.7. Quantification of Human Serum and Faecal Proteins

Blood samples were collected and processed within one hour of collection. Serum was separated from clotted blood by centrifugation at 2000× *g* for 10 min at 4 °C. Serum was stored at −80 °C until analysis.

Faecal samples were self-collected and stored at −80 °C until processing. Faecal processing was adapted from a previously described protocol [66]. Briefly, samples were thawed on ice and homogenized to a concentration of 500 mg/mL in 1 × PBS supplemented with protease inhibitor cocktail (cOmplete EDTA-free, Roche (1 tablet in 10 mL) and phenylmethylsulfonyl fluoride (0.3 mM)) to prevent protein degradation. Samples were homogenized by vigorous shaking without beads at 25 Hz for 60 s and centrifuged at 12,000× *g* for 20 min at 4 °C, and the supernatant was collected.

Serum and faecal TFF1 and TFF2 were analysed by sandwich ELISA (DuoSet ELISA kits, R&D systems, Minneapolis, MN, USA) following the manufacturer’s instructions.

### 4.8. Statistical Analysis

Preclinical data were represented as mean ± standard error of the mean (SEM). The two-tailed Student’s *t*-test was used for two-way comparisons, and one-way analysis of variance (ANOVA) with post-hoc Dunnett’s test for three or more comparisons. Human data in this study proved not to be normally distributed, so non-parametric statistical tests were applied. Data were displayed as median ± interquartile range (IQR), and the Friedman test with Dunn’s post-hoc multiple comparisons test was used for comparing paired serum or faecal concentrations. Patients with incomplete datasets were excluded from statistical analysis, i.e., when at least one blood or faecal sample was not obtained. *p* < 0.05 was considered statistically significant. Statistical analysis was performed using GraphPad Prism version 9.5.1 (GraphPad software, La Jolla, CA, USA).

## 5. Conclusions

This study has demonstrated that the exclusively small intestinal helminth, *H. polygyrus*, induces a site-specific and sustained change in the composition of colonic mucus. This includes the upregulation of goblet-cells-derived proteins and mucin sialylation, which alter the properties of mucus and shape the colonic mucus microbiome. These findings highlight another process by which helminths could influence the health of the host at sites distant to the site of infection and provide a plausible mechanism by which hookworm therapy could be used to treat ulcerative colitis.

## Data Availability

Sequence data are available at Dryad: https://datadryad.org/stash/share/NwLmRsMW0cQAwnV3hjCU-N2DKwZ-CYSCRS_9Y3ETIuI.

## Supplementary Materials

The supporting information can be downloaded at: https://www.mdpi.com/article/10.3390/ijms252212015/s1.
