# Supplementary material for: A Small Intestinal Helminth Infection Alters Colonic Mucus and Shapes the Colonic Mucus Microbiome
Source: Int J Mol Sci. 2024 Nov 8;25(22):12015. doi: 10.3390/ijms252212015 (PMC11593901; doi:10.3390/ijms252212015)
Supplement: Supplementary file 1 [file ijms-25-12015-s001.zip › ijms-3231964-supplementary.pdf]

**PCR primer sequences**		
**Primer**	**5'- Forward primer- 3'**	**5'- Reverse primer-3'**
Muc2	*GATGGCACCTACCTCGTTGT*	*GTCCTGGCACTTGTTGGAAT*
Muc5ac	ACACCGCTCTGATGTTCCTC	TCCTGGGTTGAAGGCTCGTA
Muc5b	TCACCTATGTGCTCCTCAGGG	AGGAGACCTGCACGACTGTAG
Muc6	AGCTGTGCCACACTATCTGAGT	CACTCCTGGTACACTTGGTTGG
Tff1	CCGGGAGAGGATAAATTGTGGC	AATCCCCGGACACTGTCATCA
Tff2	CTTTGACTCTAGCGTCGCTGG	CTTGCGAGCTGACACTTCCAT
Tff3	TTGCTGGGTCCTCTGGGATAG	TACACTGCTCCGATGTGACAG
St6galnac1	TAAGGCCAGCACCAGAGAAT	TGTTCTGACACAGGCGTCTT
RPLP0	AGATTCGGGATATGCTGTTGGC	TCGGGTCCTAGACCAGTGTTC
**TaqMan probes**	**Colour**	**Assay ID (ThermoFisher™)**
Relm-β	FAM	Mm00445845_m1
Gal3st2	FAM	Mm07308186_g1
Chst-4	FAM	Mm00488783_s1
St3gal3	FAM	Mm00493353_m1
GAPDH	VIC	Mm99999915_g1

Table S1 Table: Forward and reverse primer sequences, and TaqMan probes

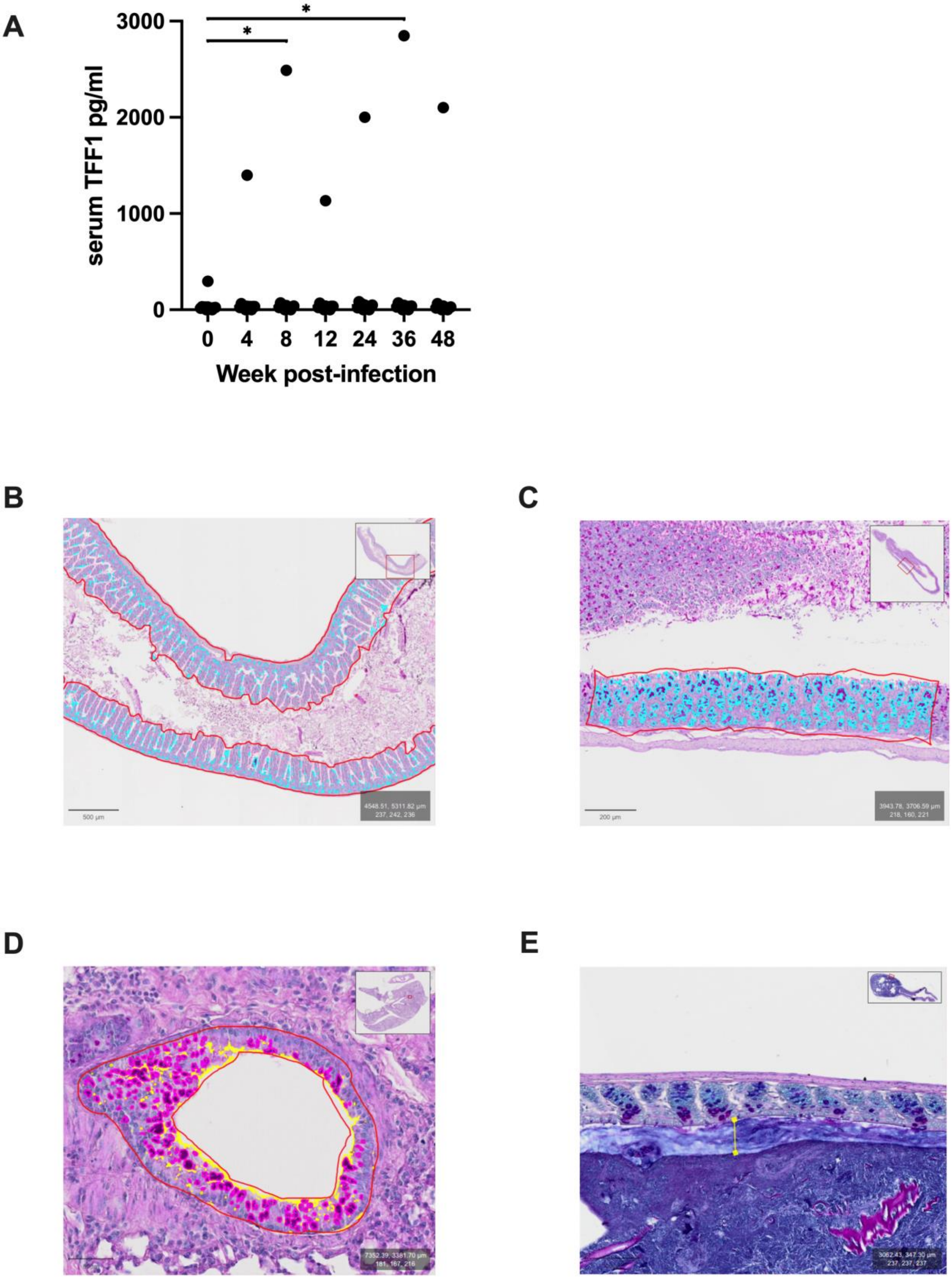

Figure S1: A) ELISA results showing concentrations of human serum TFF1 in all 12 human participants infected with human hookworm**. B), C)** and **D)** Representative histological sections of PAS staining of the jejunum (B), colon (C) and lung (D)

demonstrating selection of the epithelial layer and goblet cells used for goblet cell quantification. **E)** Representative histological section of the colon with PAS/Alcian blue staining demonstrating the measurement used to quantify mucus thickness.
